# A novel profluorescent paramagnetic diaza-crown ether: synthesis, characterization and alkaline metal-ion complexation†

**DOI:** 10.1039/c8ra09828j

**Published:** 2019-02-19

**Authors:** Anca G. Coman, Cristina Stavarache, Anca Paun, Codruţa C. Popescu, Niculina D. Hădade, Petre Ionita, Mihaela Matache

**Affiliations:** University of Bucharest, Faculty of Chemistry, Department of Organic Chemistry, Biochemistry and Catalysis, Research Centre of Applied Organic Chemistry 90-92 Panduri Street RO-050663 Bucharest Romania mihaela.matache@g.unibuc.ro pionita@icf.ro; Institute of Organic Chemistry “C.D. Nenitescu” of the Romanian Academy 202B Spl. Independentei 060023 Bucharest Romania; Faculty of Chemistry and Chemical Engineering, Supramolecular Organic and Organometallic Chemistry Centre, “Babes–Bolyai” University 11 Arany Janos Str. RO-400028-Cluj-Napoca Romania; Institute of Physical Chemistry “Ilie Murgulescu” 202 Splaiul Independentei Bucharest Romania

## Abstract

Starting from Kryptofix 22 two different branches were covalently attached through the nitrogen atoms, one containing a fluorescent moiety and the other the stable free radical TEMPO. The novel derivative exhibits fluorescence and paramagnetic properties, while the diaza-crown part ensures the affinity for alkaline metal-ions.

## Abstract 2

Starting from Kryptofix 22 two different branches were covalently attached through the nitrogen atoms, one containing a fluorescent moiety and the other the stable free radical TEMPO.

## Graphical abstract

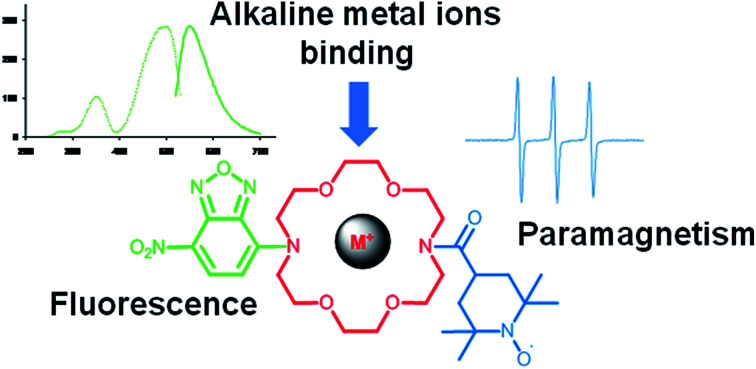

Fluorescence^1^ and electron paramagnetic resonance (EPR)^2^ are two versatile techniques that found useful applications in various fields, especially for detection purposes, taking advantage of the two very different working principles, *i.e*. fluorescence as an optical method, and EPR as a magnetic method. Combination of these two techniques is possible by coupling in the same molecule two different moieties, one fluorescent and one paramagnetic, yielding a dual behaviour of the resulted compound, usually called profluorescent free radicals.^3^ Such examples are very useful for the detection of numerous biological analytes with very interesting performance.^4^ Stable free radicals of nitroxide type are often encountered in the literature due to their great stability in an open atmosphere (they do not react with oxygen, nor dimerize) over a large range of temperatures. Usually, covalent attachment of a nitroxide moiety to a fluorescent compound leads to intramolecular fluorescence quenching. By switching off the paramagnetic centre (*i.e. via* reduction reactions), the fluorescence can be restored, providing dual paramagnetic-fluorogenic probes useful as detection tools.

Our previous work^5^ in the field of profluorescent nitroxides involved synthesis of novel compounds based on a new type of fluorogenic core, 2,5-disubstituted-1,3,4-oxadiazoles and TEMPO (2,2,6,6-tetramethylpiperidine-*N*-oxyl) stable free radical, as paramagnetic component that were demonstrated useful in the detection and quantification of some analytes, *i.e.* sodium ascorbate.

Continuing this topic, we aimed for preparation of new profluorescent nitroxides that contain a third functional unit. Aza-crown ether moieties^6^ are widely encountered in supramolecular assemblies, for synthesis of host-guest systems, generally acting as receptors for metal-ions.^7^ Combining all these structural motifs may result in unusual chemical, optical, or electronic properties, considering that aza-crown ethers covalently functionalized with fluorophores were successfully used in analytical chemistry as (chemo)sensors, due to high sensitivity of the fluorescence technique and the affinity of the aza-crown ether moiety for specific cations.^8^ Whereas fluorescent (aza)crown ethers are widely encountered in literature, spin-labelled (aza)crown ethers were much less explored; very few available papers describe, besides the expected complexation, structural and dynamical information about the environmental recognition processes.^9^

To the best of our knowledge, there is only one paper^10^ describing a double crown ether sensor, containing a fluorophore (acridine) and a paramagnetic core (nitroxide), covalently linked through a 18-crown-6 ether moiety. In this context, we describe the synthesis and characterisation of a new nitrobenzo-1,2,5-oxadiazole (NBD) and/or TEMPO functionalised compounds in which the fluorophore and free radical are linked through a diaza-crown ether (Scheme 1). We investigated the possibility to switch between luminescence and paramagnetism as well as the ability to form complexes with alkaline metal-ions. The choice for NBD as fluorogenic unit was based on its widely use for development of chemosensors for various analytes *i.e.* cysteine, homocysteine, glutathione, vitamin C.^11^ NBD-chloride is a versatile reagent that is highly reactive in S_N_Ar with common N, O or S nucleophiles, and, therefore, it was one of the first reagents used in non-specific protein labelling.^12^

**Scheme 1:**
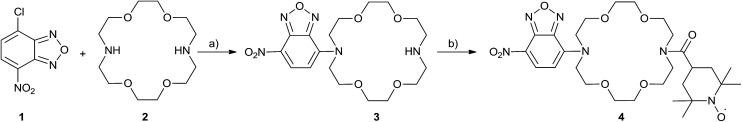
Synthesis of compounds 3 and 4: (a) DCM, Et_3_N, rt, overnight, 62%; (b) 4-carboxy-TEMPO, PyBOP, DIPEA, DCM, rt, 4h, 81%.

Synthesis of the novel fluorescent diaza-crown ether 3 was accomplished by reaction of the furazane 1 and the aza-crown ether 2, well known as Kryptofix 22, in a good yield (62%) by simply stirring the two reactants in dichloromethane (DCM), using triethylamine as base. Compound 3 has not been described up to now. However, the disubstituted aza-crown derivative was previously obtained through a slightly modified procedure.^8*b*,13^ Further, compound 3 underwent an amide coupling reaction with 4-carboxy-TEMPO, using common amide coupling conditions: activating reagent PyBOP (benzotriazol-1-yl-oxytripyrrolidinophosphonium hexafluoro-phosphate) and DIPEA (*N*,*N*-diisopropylethylamine) as base in DCM. The reaction proceeded smoothly, in a very short reaction time, in 81% isolated yield.

The NMR study (Fig. 1) of compound 3 was performed in DMSO-*d*_6_ and CDCl_3_ (see ESI† for full spectra). In the aromatic region of the spectrum registered in DMSO-*d*_6_, we could observe a downfield of the chemical shifts corresponding to the signals of the aromatic protons (*δ*_H_5__ = 8.46/8.40 ppm and *δ*_H_6__ = 6.54/6.21 ppm for DMSO-*d*_6_/CDCl_3_), whereas in the aliphatic region, the signals corresponding to the methylene protons of the ether residue are slightly shielded (*i.e. δ*_H_8/8'__ = 4.23/4.30 ppm *δ*_H_9/9'__ = 3.79/3.86 ppm for DMSO-*d*_6_/CDCl_3_). The data obtained in DMSO-*d*_6_ is consistent with previously reported data for the disubstituted derivative.^8*b*,13^ However, the high shield of the signals corresponding to the aromatic protons, especially H_6_ was intriguing. Structurally similar aza-crown ethers bearing only one nitrogen atom displayed a chemical shift corresponding to H_6_ at *δ* = 6.35 ppm.^8*b*^ Previous studies showed that the conformation of the aza-crown ethers is highly dependent on the substituents of the nitrogen atoms, which undergo inversion when substituted, with the lone pair electrons oriented toward the interior of the cavity.^14^ In this context, the high shield of the aromatic protons in the vicinity of the aza-crown ether moiety could be caused by conformation changes in environments of different polarities.

**Fig. 1:**
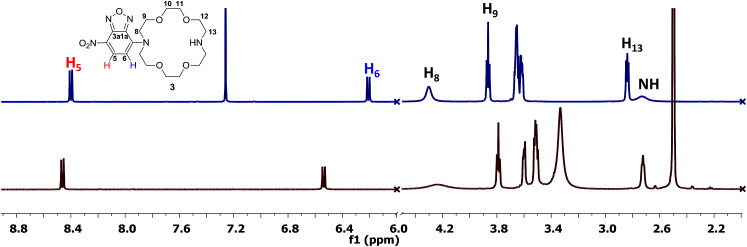
^1^H NMR spectra (fragments, 500 MHz) of compound 3 in CDCl_3_ (top) and DMSO-*d*_6_ (bottom).

Another interesting observation with respect to previous reported data is the value of the chemical shifts corresponding to protons H_8/8'_ and H_9/9'_ which are significantly downfield compared to similar compound bearing *N*-methyl groups (*δ* = 2.74 ppm and *δ* = 3.56 ppm).^14^ A possible hindered rotation between the aza-crown ether and the *p*-nitrofurazane moieties may also be the cause of the signals broadening. This could be observed for the signal corresponding to H_8_ (*δ* = 4.23 ppm, broad signal). The carbon spectrum also displays broad signals of the carbons labelled with C-8 and C-9 (*δ* = 53.6 ppm and *δ* = 68.6 ppm, respectively, see ESI†). Finally, the proton of the free nitrogen atom is visible in the spectrum registered in CDCl_3_, at *δ* = 2.67 ppm, in accordance to previously reported data for similar compounds, although slightly downfield shifted.^15^

The absorption spectrum of compound 3 registered in DMSO at 20 μM (Fig. 2) showed absorption maxima around 350 and 500 nm (Table 1), corresponding to the transitions of the NBD moiety,^13^ indicating that attachment of the diaza-crown ether moiety did not influence the absorption behaviour of the oxadiazole. Variation of absorption maxima according to the solvent used, as inferred from the literature data^8*b*^ confirm our results and the slight solvatochromic behaviour of the NBD-derived compound.

**Fig. 2:**
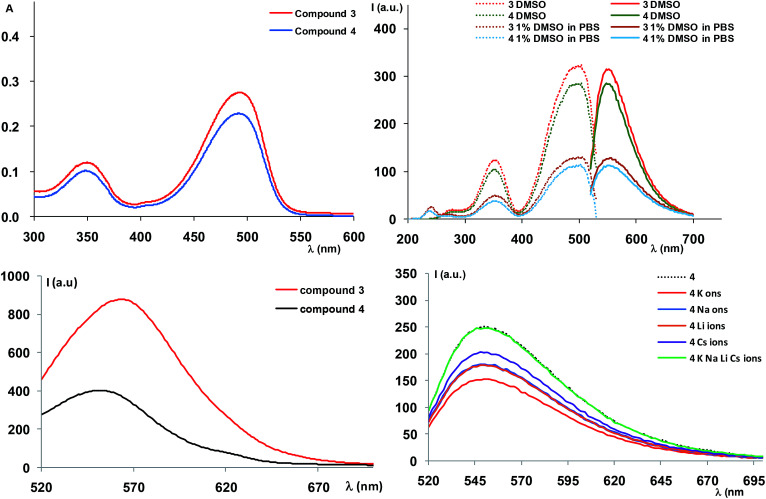
Top: Left – absorption spectra of compounds 3 and 4, recorded in DMSO at 2 × 10^−5^ M. Right: Excitation (dotted lines) and emission (plain lines) of compounds 3 and 4, recorded in DMSO and 1% DMSO in PBS buffer at 10^−4^ M. Bottom: Left – solid state emission spectra of compounds 3 and 4 (*λ*_ex_ = 490 nm). Right – emission spectra (*λ*_exc_ = 500 nm) of compound 4 with aqueous solutions (MiliQ water) of LiClO_4_, NaClO_4_, KClO_4_ and CsClO_3_ (individual and equimolar amounts) at final concentration of the organic compounds of 10^−4^ M.

**Table 1:** Absorption and emission wavelength for compounds 3 and 4 in solution and solid state

Cmp	Solvent	*λ*_abs_ (nm)	*λ*_exc_ (nm)	*λ*_em_ (nm)	Stokes shift (cm^−1^ × 10^−3^, nm)
**Solution**					
3	DMSO	345, 493	348, 500	550	1.818, 50
	1% DMSO in PBS	342, 500	342, 500	554	1.819, 54
4	DMSO	344, 492	350, 500	548	1.817, 48
	1% DMSO in PBS	354, 500	354, 500	550	1.818, 50

**Solid state**					
3			490	564	2677, 74
4			490	552	2292, 62

Further measurement of the fluorescence spectra also showed a variation of the luminescence intensity according to the solvent polarity. Thus, the spectra recorded in DMSO or mixture of DMSO/water (1% DMSO in PBS buffer or 10% DMSO in ultrapure water) displayed emission maxima at *λ*_em_ = 550 nm (*λ*_ex_ = 500 nm), corresponding to the NBD moiety emission. However, the intensity of the emission bands decreased upon polarity increase, suggesting fluorescence quenching as a result of the polar solvent induced aggregation of the organic molecules.

Solid state fluorescence of compound 3 (Fig. 2) indicated an emission maximum at *λ*_em_ = 564 nm (*λ*_ex_ = 490 nm), slightly red shifted compared to the emission in solution (Table 1), with an appreciable Stokes shift (74 nm).

We further studied the behaviour of compound 3 in presence of alkaline metal ions (Li^+^, Na^+^, K^+^) by NMR, in order to assess the ability of the new compound for complexation. As previously mentioned, the nitrogen lone pair electrons were demonstrated to afford a better binding when substituents are present.^14^ However, most of the examples include aliphatic substituents^8*b*,14^ while in our case conjugation occurs by coupling the aza-crown ether to the aromatic NBD moiety.

Thus, addition of excess amounts of KClO_4_ in D_2_O to a solution of 3 in DMSO-*d*_6_ (approx. 25 mM final concentration) led to more visible changes in the ^1^H NMR spectra profiles, compared to addition of lithium or sodium ions (Fig. 3 and ESI†). This could be correlated with the dimensional fit between the size of the crown-ether cavity and the diameter of the cation, suggesting a higher affinity of the compound for potassium ions.^16^ However, the chemical shifts slightly changed also for the two other cations, indicating a non-selective behaviour of 3. Generally, the signals were well resolved and could be assigned to most protons in the structure of compound 3. The signal multiplicities preserved upon addition of the aqueous metal ion solution and we could notice a shield of the aromatic doublets with approximately Δ*δ* = 0.13 ppm for the proton in the vicinity of the nitro group (H_5_, *δ* = 8.46 ppm, *δ*' = 8.33 ppm) and Δ*δ* = 0.21 ppm for the proton in the vicinity of the crown-ether moiety (H_6_, *δ* = 6.53 ppm, *δ*' = 6.32 ppm) upon addition of potassium ions solution (Fig. 3). Significant changes were also visible in the aliphatic region: all signals of the mixtures were shielded. For example, the protons next to the conjugated nitrogen atom (H_8_, *δ* = 4.23 ppm, *δ*' = 4.14 ppm) yield a broad signal, which shifts with approximately Δ*δ* = 0.08 ppm. Spectra containing only deuterated water and no metal ion were also recorded to confirm that the changes in the spectra were the effect of the alkaline metal ions. The unusual shielding effect could be explained by changes in the conformation of the compound upon complexation, as also previously observed by others in studies regarding behaviour of diazacrown ethers toward metal ions like barium.^17^ We have also performed titration experiments with potassium ions in DMSO-*d*_6_ and confirmed 1 : 1 stoichiometry (see ESI†). However, the association constant was found to be rather low (<10^2^ M^−1^, see ESI†), suggesting that the nitrogen atom contributes less, due to involvement in conjugation with the aromatic system.^18^

**Fig. 3:**
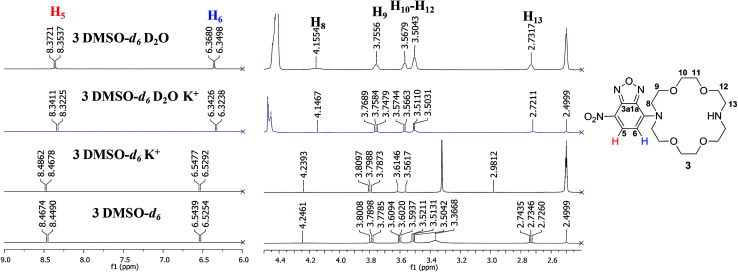
^1^H NMR spectra (500 MHz, fragments) of 3, (25 mM in DMSO-*d*_6_, 500 MHz) and mixture of 3 with KClO_4_ (5 fold excess) in D_2_O or DMSO-*d*_6_.

Complexation studies were also performed by ESI(+)-MS experiments. The ability of compound 3 to host alkaline metal ions was assessed by running experiments with alkaline metal ions (LiClO_4_, NaClO_4_, KClO_4_ and CsClO_3_), indicating formation of all supramolecular complexes between compound 3 and each of the tested alkaline metal ions (see ESI† for full spectra).

Once compound 3 characterized, we turned our attention to the triple functional compound 4 and investigated the paramagnetic properties, optical behaviour and complexation properties. Thus, the EPR spectrum showed the expected triplet of a nitroxide free radical (Fig. 4), with a hyperfine coupling constant of 1.575 mT. The intensity of the high field line is slightly smaller, confirming the successful attachment of the free radical moiety to the diaza-crown ether central unit. The HRMS spectrum also confirms the formation of the target structure (see ESI†).

**Fig. 4:**
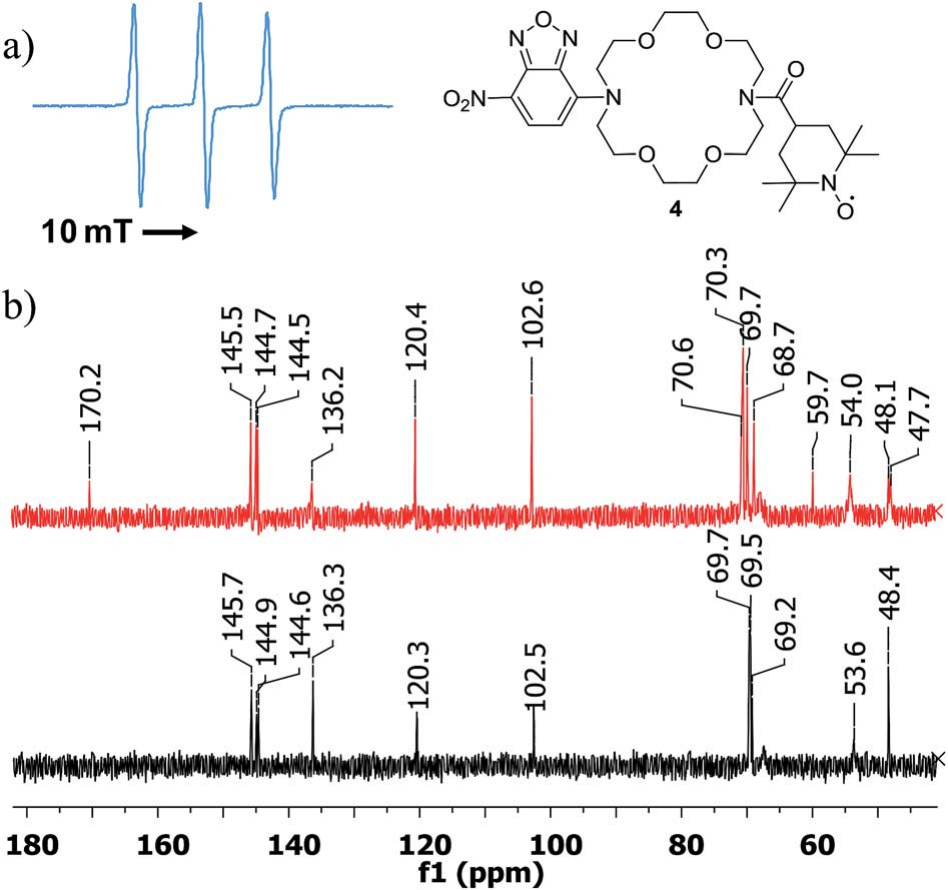
(a) EPR spectrum of compound 4, registered in DCM. (b) ^13^C NMR spectra (fragments) of 4 (top) and 3 (bottom).

Recently,^19^ TEMPO-derived compounds were investigated by ^1^H and ^13^C NMR experiments, indicating that carbon spectra can be very informative for the atoms located far enough from the free radical site. This was also confirmed in our case (see ESI† for full spectra and Fig. 4). While the proton spectrum displays broad, unresolved signals, the ^13^C NMR spectrum of compound 4 indicated signals that could be assigned to the furazan and diaza-crown ether cores as well as the carbonyl carbon from the amide moiety at *δ* = 170.2 ppm. In addition, the spectrum showed signals that seem to correspond to carbons of the TEMPO skeleton. However, precise assignment could not be performed, due to lower solubility of our compound (unlike previous results, which were possible using very high concentrations) and low accuracy of the 2D NMR experiments. In addition, the stability of the compound in DMSO-*d*_6_ seems to be affected in time, making the analysis of lengthy experiments to be less reliable.

In a recent paper^20^ Lucarini *et al.* demonstrated the use of a novel spin-labelled crown-ether in sensing of metal and organic cations, in host-guest complexation processes. Measurements were based on the change that occurs on EPR hyperfine splitting constants of benzylic hydrogen and/or of nitrogen atom from nitroxide moiety. In our case, only nitrogen hyperfine splitting constants might be affected, but test experiments showed no change outside the experimental errors.

Investigation of compound 4 by ESI(+)-MS revealed a profile of the mass spectrum that is coherent with previous studies of nitroxide based free radicals.^21^ Various redox processes may occur under the ionisation conditions leading to fragmentation of the free radical moiety (see ESI† for full spectrum). Thus, the resulted base peak (*m*/*z* = 593.3257) should correspond to species [M + H_2_–O]^+^, previously observed^21^ for TEMPO-based radicals. In addition, a peak at *m*/*z* = 609.3207 (14%) corresponding to [M + H_2_]^+^ ion could have resulted from the protonated hydroxylamine *via* a reduction process. The spectrum also shows the [M + Na]^+^ ion peak (630.2946, 27%). Complexation studies with alkaline metal ions showed the expected host guest complexes for all cations (LiClO_4_, NaClO_4_, KClO_4_ and CsClO_3_). The competitive experiments using solutions containing equimolar amounts of the three metal ion salts, in five fold excess, yielded peaks with different relative abundances: base peak for [M + Li]^+^ (614.3284), 68% for [M + Cs]^+^ peak (740.2188), 23% for [M + Na]^+^ (630.3020) and 18% for [M + K]^+^ peak (646.2761). All these data suggest that compound 4 acts as a non-selective host for the alkaline metal-ions.

We further set to study the potent profluorescent behaviour. The absorption spectra registered in solution in the same conditions as for compound 3 indicated a similar profile, caused by the fluorogenic NBD moiety (Fig. 2). The fluorescence spectra also behaved similarly in terms of profiles in organic solvent and water, namely we noticed a decrease in the luminescence intensity by increase in the solvent polarity (Fig. 2). However, a much more interesting observation was the decreased fluorescence intensity of approximately 20% with respect to precursor 3 which suggested that the fluorophore did not strongly interact with the free radical system, in order to cause complete luminescence quench, as in the case of our previously described compounds.^5^ This could be mainly attributed to the lack of a conjugated system which usually affords complete switching cycles between the paramagnetic and fluorescent states, upon applying a redox trigger. In addition, in solid state (Fig. 2) we could observe a significant quench of the fluorescence, along with a blue shift of the emission maximum (*λ*_em_ = 552 nm, *λ*_ex_ = 490 nm, Table 1), suggesting stronger interactions between fluorophore and free radical and the profluorescent character of 4.

In fluorescence studies (Fig. 2), we observed that addition of fivefold excess of alkaline metal ions (LiClO_4_, NaClO_4_, KClO_4_ and CsClO_3_) to compound 4 led to decrease in the fluorescence intensity for all metal ions. The experiments were preformed in triplicates and were reproducible. The effect of water over the fluorescence quench was negligible (we used aprox. 2% water in DMSO, which has limited effect on the fluorescence) The fluorescence quench was more significant for potassium ions, especially with respect to the same experiments performed for the precursor 3 (see ESI†). This might be an indication of some interactions that occur between the fluorophore unit and the free radical that are mediated by metal ions. However, the EPR spectra of the mixtures between compound 4 and metal-ions do not show any major change. Use of heavy metals or other solvents could bring other interesting observations and we are currently expanding our studies.

## Conclusions

In conclusion, we described the synthesis of new fluorophore-aza-crown-ether and fluorophore-aza-crown-ether-free radical conjugates and their structural investigation, photophysical properties and behaviour in presence of alkaline metal ions. The collected data suggested a weak interaction between the fluorophore and the free radical (visible from preservation of the luminescence when the free radical is active) in the fluorophore-crown-ether-free radical conjugate. On the other hand, complexation experiments with the alkaline metal ions (Li^+^, Na^+^, K^+^, Cs^+^) was studied by NMR and ESI(+)-MS, indicating the ability of the compounds to form supramolecular complexes, with a slight preference for potassium ions. Partial quench of fluorescence in solid state or by addition of metal ions may be further exploited and such multifunctional systems containing: (i) an aza-crown ether residue, (ii) a paramagnetic component and (iii) a fluorophore could find useful applications as sensors for monitoring many processes that occur in (bio)chemical systems (*i.e.* spin-probes, markers, imaging).

## Conflicts of interest

There are no conflicts to declare.

## Supplementary Material

RA-009-C8RA09828J-s001
