# Supplementary material for: A novel profluorescent paramagnetic diaza-crown ether: synthesis, characterization and alkaline metal-ion complexation
Source: RSC Adv. 2019 Feb 19;9(11):6078–83. doi: 10.1039/c8ra09828j (PMC9060903; doi:10.1039/c8ra09828j)
Supplement: RA-009-C8RA09828J-s001 [file RA-009-C8RA09828J-s001.pdf]

Electronic Supplementary Material (ESI) for RSC Advances.
This journal is © The Royal Society of Chemistry 2019

Supporting Information

for

A novel profluorescent paramagnetic diaza-crown ether: synthesis, characterization and alkaline metal-ions complexation

Anca G. Coman,[a] Cristina Stavarache,[b] Anca Păun,[a] Codruța C. Popescu,[a] Niculina D. Hădade,[c] Petre Ionita*[a,d] and Mihaela Matache*[a]

[a] *University of Bucharest, Faculty of Chemistry, Department of Organic Chemistry, Biochemistry and Catalysis, Research Centre of Applied Organic Chemistry, 90-92 Panduri Street, RO-050663 Bucharest, Romania; e-mail: mihaela.matache@g.unibuc.ro (M. Matache); pionita@icf.ro (P. Ionita)*

[b] *Institute of Organic Chemistry "C.D. Nenitescu" of the Romanian Academy, 202B Spl. Independentei, 060023, Bucharest, Romania*

[c] *Faculty of Chemistry and Chemical Engineering, Supramolecular Organic and Organometallic Chemistry Centre, ''Babes--Bolyai'' University, 11 Arany Janos Str., RO-400028-Cluj-Napoca, Romania;*

[d] *Institute of Physical Chemistry "Ilie Murgulescu", 202 Splaiul Independentei, Bucharest, Romania*

1. Synthesis

General information. All solvents and reagents purchased from commercial suppliers were used without further purification. Thin layer chromatography (TLC) was performed on silica gel 60 coated aluminium F254 plates with visualization by UV irradiation at 254 and 365 nm. The NMR spectra were recorded on Bruker Advance Ultrashield Plus spectrometer operating at 500 MHz for ^{1}H and 125 MHz for ^{13}C. High resolution mass spectra were recorded on a Thermo Scientific (LTQ XL Orbitrap) spectrometer, in positive ion mode, using ESI-(+) technique. Melting points were determined in open capillary tubes using a STUART SMP3 electric melting point apparatus and are uncorrected. The UV-Vis spectra were recorded a Jasco V-630 spectrophotometer using 10 mm quartz cell. The solution fluorescence spectra were performed on a Thermo Scientific Varioskan Flash spectral scanning multimode reader. Electron paramagnetic resonance (EPR) spectra were recorded on a Jeol Jes FA 100 apparatus.

4-nitro-7-(1,4,10,13-tetraoxa-7,16-diazacyclooctadecan-7-yl)benzo[c][1,2,5]oxadiazole 3.

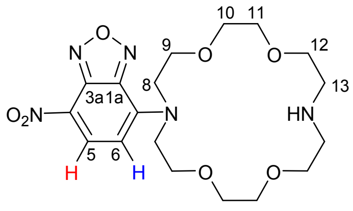

3

Dark-red solid. Yield 62 % (0.260 g) *m.p.* 83-84°C. R_f=0.19 (silica, DCM:MeOH=9:1). To a solution of **2** (1.1 mmol) in DCM (50 mL) was added dropwise, under stirring, in a few hours, a solution of **1** (1 mmol) in DCM (50 mL). The reaction mixture was left to react overnight, then triethylamine (1 mL) was added and left under stirring overnight. The solvent was evaporated under vacuum and the compound was purified by column chromatography (silicagel, ethyl acetate).

^{1}H NMR (500,13 MHz, DMSO-d_6): δ= 8.46 (d, 1H, ^{3}J=9.2 Hz, H-5); 6.54 (d, 1H, ^{3}J=9.3 Hz, H-6); 4.23 (bs, 4H, H-8, H-8'); 3.79 (t, 4H, ^{3}J=5.6 Hz, H-9, H-9'); 3.61÷3.59 (m, 4H, H-10, H-10'); 3.53÷3.50 (m, 8H, H-11, H-11', H-12, H-12'); 2.72 (t, 4H, ^{3}J=4.2 Hz, H-13, H-13') ppm. **^{13}C NMR (125,03 MHz, DMSO-d_6):** δ= 145.7 (C-4); 144.9 (C-3a); 144.6 (C-7); 136.3 (C-5); 120.4 (C-1a); 102.6 (C-6); 69.6; 69,4; 69.2 (C-12, C-12' or C-11, C-11' or C-10, C-10');67,5 (bs, C-9, C-9'); 53.6 (bs, C-8, C-8'); 48.4 (C-13, C-13') ppm.

^{1}H NMR (500,13 MHz, $CDCl_3$): *δ*=8.40 (d, 1H, ^{3}J=9.1 Hz, H-5); 6.21 (d, 1H, ^{3}J=9.1 Hz, H-6); 4.30 (bs, 4H, H-8, H-8'); 3.86 (t, 4H, syst. A_2B_2, ^{3}J =5.4 Hz, H-9, H-9'); 3.67-3.61 (m, 12H, H-10, H-11, H-10', H-11', H-12, H-12'); 2.85 (t, 4H, H-13, H-13); 2.67 (bs, H-N, deuterable) ppm. **^{13}C NMR (125,03 MHz, $CDCl_3$):** *δ*=145.3 (C-4); 144.9 (C-3a); 144.6 (C-7); 135.3 (C-5); 122.2 (C-1a); 101.2 (C-6); 70.2, 69.9, 69.8 (C-12, C-12' or C-11, C-11' or C-10, C-10'); 68.6 (bs, C-9, C-9'); 53.9 (C-8, C-8'); 49.1 (C-13, C-13').

HRMS (ESI, +) m/z: calc. for $C_{18}H_{28}N_5O_7$ $[M+H]^+$ 426.1989, found 426.1960.

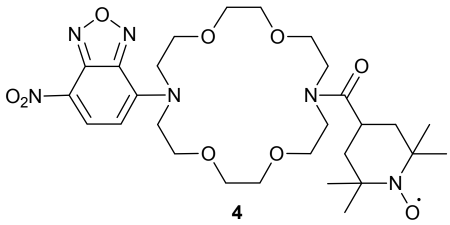

4

Nitroxide 4. Orange solid. Yield 81% (0.116g). *m.p.* 62-65°C. R_f=0.15 (silica, EtOAc). 4-Carboxy-2,2,6,6-tetramethylpiperidinyloxy (0.258 mmol) was dissolved in DCM (5 mL) and PyBOP (0.258 mmol), DIPEA (0.705 mmol) was added. Compound **3** (0.235 mmol) was dissolved in DCM (2 mL) and added to the reaction mixture. After 4h the solvent was removed under vacuum and the resulted residue was dissolved in ethyl acetate and washed with water (10 mL). The organic layer was dried over magnesium sulphate and evaporated under vacuum. The purified compound was obtained by column chromatography.

^{13}C NMR (125,03 MHz, DMSO-*d$_6$*): δ= 170.2 (C=O); 145.5 (C-4); 144.7(C-3a); 144.5 (C-7); 136.2 (C-5); 120.4 (C-1a); 102.6 (C-6); 70.6; 70.3; 69.7 (C-12, C-12' or C-11, C-11' or C-10, C-10'); 68.7 (bs, C-9, C-9'); 59.7; 53.9 (C-8, C-8'); 48.1 (C-13, C-13'); 47.8; 28.9; 21.9; 20.7; 13.9 ppm.

HRMS (ESI, +) m/z: calc. for $C_{28}H_{43}N_6O_9$ $[M]^+$ 607.3092; found 593.3257 (100%) $[M+H_2\text{-}O]^+$, 609.3207 (14%) $[MH_2]^+$ 630.2946 (27%) $[M+Na]^+$.

2. Electron paramagnetic resonance spectroscopy

Electron paramagnetic resonance (EPR) spectra were recorded on a Jeol Jes FA 100 apparatus. Solution of free radical at 10^{-4} M concentration was charged into capillary glass tube and the spectrum was recorded at room temperature. The following settings were used: frequency 8.99 GHz, centre field 322 mT, sweep width 10 mT, sweep time 120 s, time constant 30 ms, gain 200, modulation frequency 100 kHz, modulation width 0.1 mT.

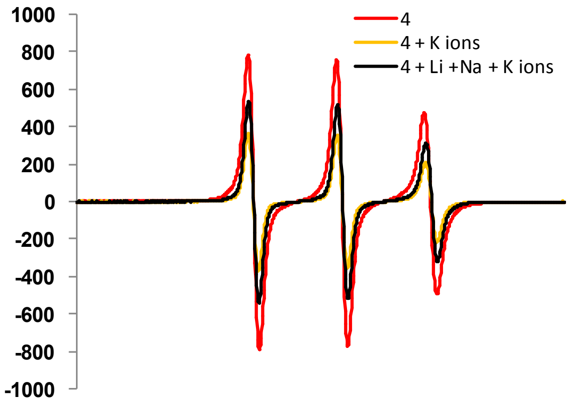

Figure S 1 Stacked EPR spectra (registered in DMSO) of compound **4** and mixtures of compound **4** and potassium ions or equimolar amounts of lithium, sodium and potassium ions

3. Absorption and fluorescence spectroscopy

a) UV-Vis spectra

Absorption spectra were recorded with a Jasco V-630 spectrophotometer, using 10 mm quartz cell. Stock solutions of compound were prepared in DMSO at 10^{-2} mol L^{-1} and diluted at 10^{-5} mol L^{-1}.

b) Fluorescence spectra

Fluorescence spectra were recorded with a Thermo Scientific Varioskan Flash spectral scanning multimode reader. The spectra were recorded in suitable plates using 5 nm excitation and emission slits for all measurements. Stock solutions of compounds were prepared in DMSO at 10^{-2} mol L^{-1} or10^{-3} mol L^{-1} and diluted to 10^{-4} mol L^{-1} in DMSO, PBS buffer pH = 7.4 or MiliQ water. All experiments were performed in triplicates and the results were averaged. The effect of water was checked and the final complexation experiments were performed using as reference solution of compound **3** the amount of water (aprox. 2% water in DMSO) that did not significantly affect the fluorescence intensity, so that any decrease of fluorescence was caused by metal ions. The same experiments were also performed for compound **4**.

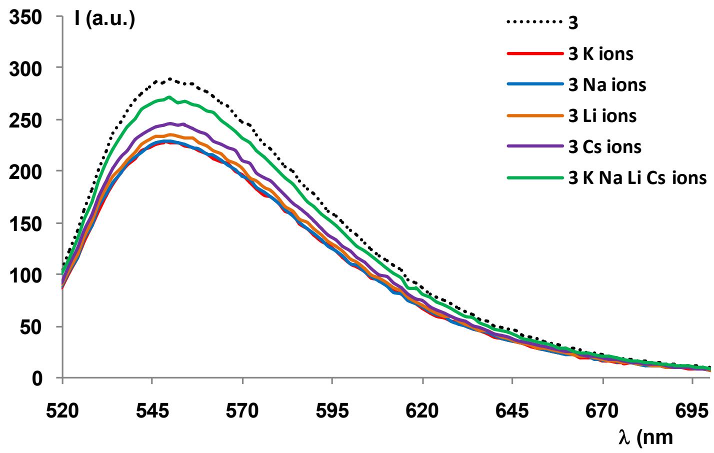

Figure S 2 Emission spectra (λ_{exc}=500 nm) of compound **3** and mixtures with aqueous solutions (MiliQ water) of $LiClO_4$, $NaClO_4$, $KClO_4$ and $CsClO_3$ (individual and equimolar amounts) at final concentration of the organic compounds of 10^{-4} M

5. Complexation studies of compound 3 with alkaline metal ions by NMR

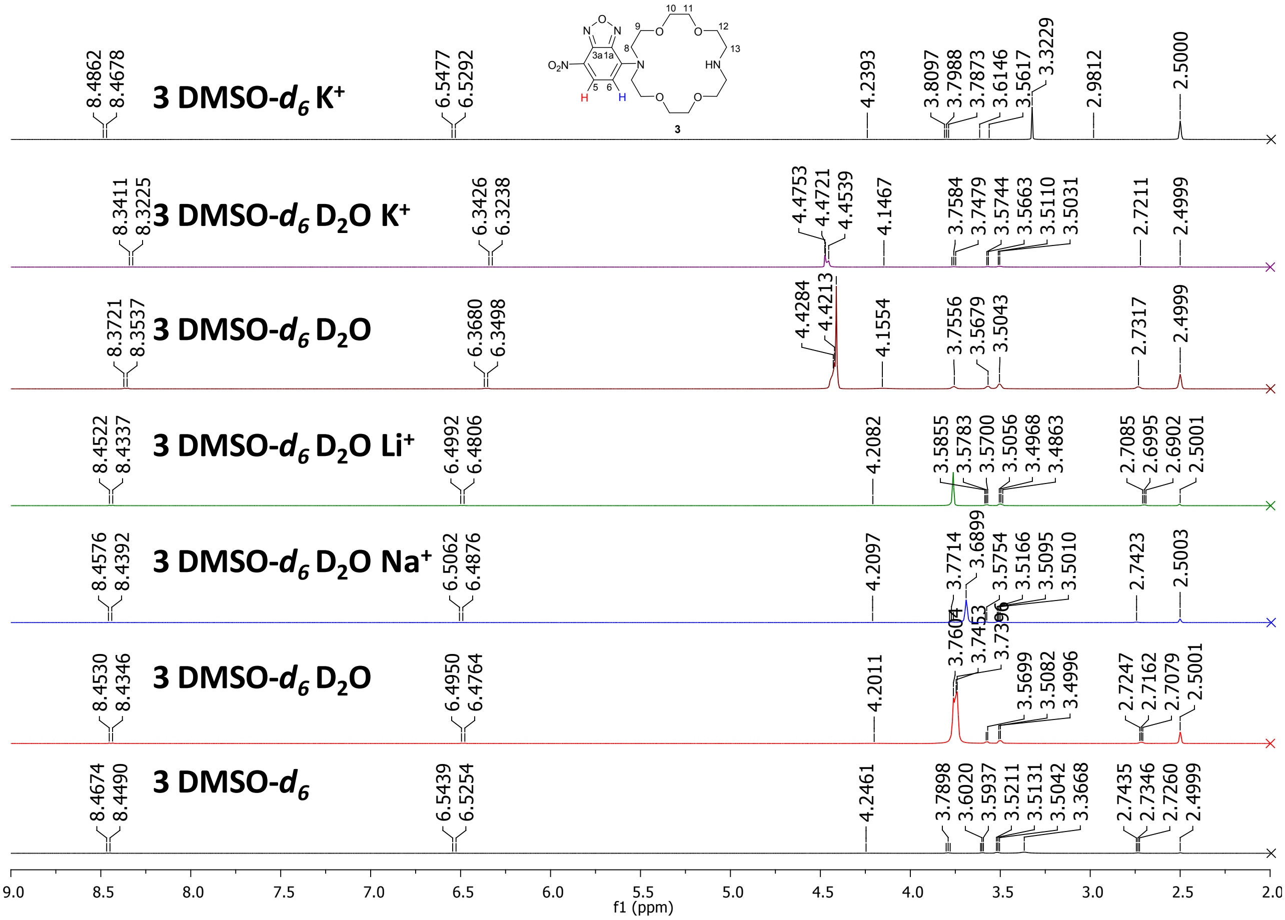

	H5	H6	H8	H9	H10	H11;H12	H13	D_20
**3** + K^+ (DMSO-d_6)	8.4769	6.53845	4.2393	3.7988	3.6281-3.6090	3.5781-3.5560	2.9874	3.32
**3** (DMSO-d_6) + K^+ (D_2O)	8,3318 (d)	6,3332 (d)	4,1467 (bs)	3,7594 (t)	3,585-3,5663 (m)	3,510-3,485 (m)	2,7211 (t)	4,48-4,45 (m)
**3** + D_2O (for K^+)	8,3629 (d)	6,3589 (d)	4,1554 (bs)	3,7656 (bs)	3,5679 (bs)	3,50 (bs)	2,7317 (t)	4,43-4,41 (m)
**3** (DMSO-d_6) + Li^+ (D_2O)	8,44295 (d)	6,4899 (d)	4,2082 (bs)	3,76 (bs) Overlapping with D_2O	3,5855 ÷ 3,570 (m)	3,5056÷3,4853 (m)	2,6996 (t)	3.77
**3** (DMSO-d_6) + Na^+ (D_2O)	8,4484 (d)	6,4969 (d)	4,2097 (bs)	3,7717 (t)	3,59-3.5754 (bs)	3,5166-3,5010 (m)	2,7423 (t)	3,69 (bs)
**3** + D_2O (for Na^+ and Li^+)	8,4438 (d)	6,4857 (d)	4,2011 (bs)	3,7604-3,74 (m) Overlapping with D_2O	3,585-3,5699 (m)	3,5082-3,485 (m)	2,7162 (t)	3.75
**3** (DMSO-d_6)	8,4582 (d)	6,53465 (d)	4,2461 (bs)	3,7898 (t)	3,6094÷3,5937 (m)	3,5211-3,5042 (m)	2,7346 (t)	3,73 (bs)

5. Titration experiments for determination of the stoichiometry between compound 3 and K^+

The stoichiometry and association constant for the formation of the complex between compound **3** and $\mathbf{K^+}$ was determined by global non-linear regression analysis of the ^{1}H NMR titration data, available through the website http://supramolecular.org.[1] Thus, we performed various host:guest mixtures, using stock solutions of **3** (7.84 mM) and $KClO_4$ (7.22 mM) in DMSO-d_6 and recorded the ^{1}H NMR spectra, following the changes of the chemical shifts at δ=3.8006 ppm, corresponding to the triplet assigned to C-9 (methylene next to an oxygen atom). Fit of the data indicated the formation of 1:1 stoichiometric complex and an association constant of 40.68 ± 26.1782 M^{-1}.

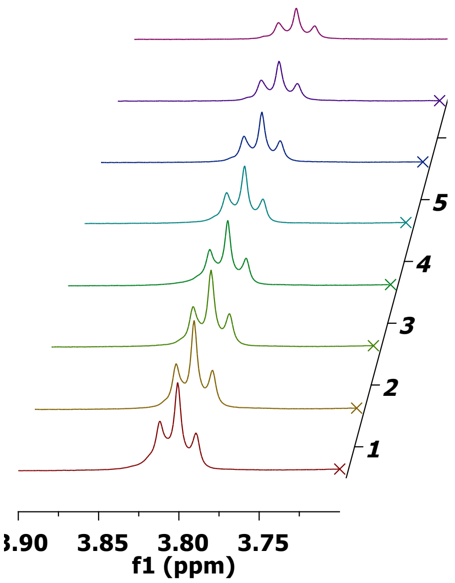

[**compound 3**] (M)	[$\mathbf{KClO_4}$] (M)	δ (ppm) H-9
0.0015686	0.0057733	3.7987
0.0023529	0.0050517	3.7991
0.0031373	0.00433	3.7996
0.0039216	0.0036083	3.7999
0.0047059	0.0028867	3.8003
0.0054902	0.002165	3.8005
0.0062745	0.0014433	3.8005
0.0070588	0.0007217	3.8008
0.0078431	0	3.8006

Figure S 3 ^{1}H NMR spectra (zoom on the reference signal at δ=3.8006 ppm, corresponding to the triplet assigned to C-9 (methylene next to an oxygen atom) and the experimental data of the NMR titration

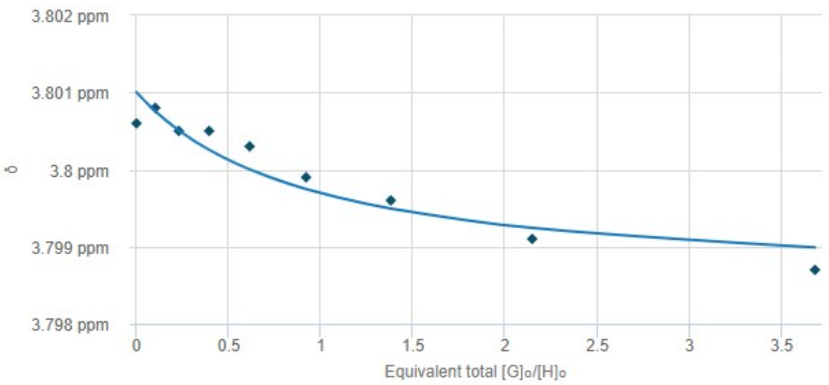

Figure S 4 Plot of the data for the titration of compound **3** with $KClO_4$ indicating 1:1 stoichiometry.

6. ^{1}H NMR spectra

[1](a) Thordarson, P. *Chem. Soc. Rev.* **2011**, *40*, 1305-1323; (b). Hibbert, D. B; Thordarson, P. Chem. Commun. **2016**, 53, 12792-12805.
